# Living with non‐cardiac chest pain – An inductive qualitative interview study of spouses' perspectives

**DOI:** 10.1002/nop2.2189

**Published:** 2024-05-25

**Authors:** Magda Eriksson‐Liebon, Anita Kärner Köhler, Peter Johansson, Ghassan Mourad

**Affiliations:** ^1^ Department of Health, Medicine and Caring Sciences Linköping University Linköping Sweden; ^2^ Department of Emergency Medicine in Norrköping and Department of Biomedical and Clinical Sciences Linköping University Norrköping Sweden; ^3^ Department of Internal Medicine in Norrköping and Department of Health, Medicine and Caring Sciences Linköping University Norrköping Sweden

**Keywords:** content analysis, experiences, non‐cardiac chest pain, qualitative interviews, spouses

## Abstract

**Aim:**

To explore spouses’ experiences of living with a partner suffering from non‐cardiac chest pain (NCPP).

**Design:**

An inductive qualitative study.

**Methods:**

Individual interviews (*n* = 10) were performed with spouses of partners having NCCP and cardiac anxiety. The analysis was performed according to Patton's guide for content analysis of qualitative data.

**Results:**

Three categories and seven subcategories were identified. First, ‘a feeling of being neglected’, where spouses felt ignored by healthcare professionals and excluded by their partners. Secondly, ‘a tension between hope and despair’ encompassed feelings of faith, support, unpreparedness for chest pain and situational frustration. Lastly, in ‘a threat to ordinary life’, spouses noted chest pain‐induced changes impacting daily life, finances, leisure and relationships. To conclude, NCCP in partners significantly affects their spouses emotionally and practically. Spouses felt neglected and isolated, oscillating between hope and despair and experiencing faith, powerlessness and frustration. They also faced challenges in daily life and relationships.

## 1 INTRODUCTION

It is well known that recurrent non‐cardiac chest pain (NCCP) has a negative impact on patients' lives, including functional impairment, impaired quality of life and psychological distress (such as cardiac anxiety, depressive symptoms and fear of bodily sensations) (Mourad et al., 2020). The pain has also a negative impact on patients' everyday life, including repeated sick leave from work and increased healthcare costs. Furthermore, the lack of explanation regarding the cause of chest pain leads to psychological distress, which drives patients to seek care (Mourad et al., 2013). Compared to patients with cardiac pain, those with NCCP report less understanding of their chest pain due to unmet information needs and loss of control over their situation (Jonsbu et al., 2012). They need healthcare professionals to pay attention by recounting their individual history of pain, showing concern for their daily lives and giving them the opportunity to ask questions (Røysland et al., 2013). Moreover, they need support in a person‐centred way based on their expectations and expressed needs.

Engagement with important others, such as spouses has shown to be of importance among patients treated for psychological distress and can result in feelings of belonging, of being understood and acknowledged and can motivate patients to engage in their treatment (Wilhelmsen et al., 2013). However, despite many years of research on patients with NCCP (Mourad et al., 2022; Mulder et al., 2019), no studies have to our knowledge been conducted to explore spouses´ experiences of seeing their partners live with NCCP or spousal engagement in their partners' treatment.

## 2 BACKGROUND

There is a lack of studies regarding how NCCP impacts the lives of patients' relatives; however, research shows that living with long term, chronic pain is challenging for patients and their families (Gjesdal et al., 2018). Spouses of patients with chronic illnesses including chronic pain, have been shown to suffer from psychosocial burden and loneliness and to struggle with feelings like anger, guilt, helplessness, worry, lost faith in the future and going unrecognized or unacknowledged by healthcare professionals (Chistell et al., 2023; Montesó‐Curto et al., 2022). The family plays an important role in the management of chronic pain and their involvement should be balanced between what is feasible and beneficial for the couple (Brown & Newton‐John, 2023; Swift et al., 2014). However, interactions between the family and the patient with chronic pain are usually complex, as pain has been shown to have an impact on all aspects of their everyday life, which results in changes for family members. Consequences of different characters, such as reduced social interaction, limitations in sexual life, reduced career opportunities and financial loss and uncertainty about the future, engaging in escape‐avoidance coping in order to deal with the demands of caregiving. All together, this results in cumulative negative changes to the family's life (Granero‐Molina et al., 2023; Montesó‐Curto et al., 2022). Hence, spouses of patients with NCCP, where no medical diagnosis has been established, may live with similar experiences. To our knowledge, however, no previous studies have explored how spouses of patients with NCCP feel that their lives are affected by their partner's chest pain.

## 3 THE STUDY

### 3.1 Aim and objective

The aim of this study was to explore spouses’ experiences of living with a partner suffering from NCCP. The objective is to gain an understanding of spouses' experiences, which is crucial for healthcare professionals to better support patients and their spouses dealing with NCCP.

## 4 METHOD

### 4.1 Design

This was a qualitative and exploratory interview study with an inductive approach. This study is part of a project that aimed to evaluate the effects of a nurse‐led internet‐based cognitive behavioural therapy (iCBT) program on cardiac anxiety and other patient‐reported outcomes, such as fear of bodily sensations, depressive symptoms, health‐related quality of life and chest pain frequency in patients with NCCP. Study data was collected through interviews with spouses to partners participating in the aforementioned iCBT study.

### 4.2 Participants and recruitment

Inclusion criteria were: Swedish‐speaking spouses to patients with NCCP and psychological distress who had participated in the main randomized controlled study and had been randomized to the iCBT group (Mourad et al., 2022). There were no exclusion criteria. After receiving permission from participants in the iCBT group, a random sample was employed, in which all spouses were asked to participate until the COVID‐19 pandemic stopped data collection as physical interviews were no longer possible to perform. In total, 31 spouses were invited to participate in the study and 10 agreed to participate and were interviewed. The characteristics of the participants are presented in Table 1.

**TABLE 1:** Participant characteristics (*N* = 10).

Characteristics	
**Sex, *n* (%)**	
Females	6 (60)
Males	4 (40)
**Age, year (mean ± SD)**	61 ± 15
**Marital status, *n* (%)**	
Married	7 (70)
In relationship	3 (30)
**Place of residence, *n* (%)**	
Urban	4 (40)
Rural	6 (60)
**Occupational status, *n* (%)**	
Working	6 (60)
Retired	4 (40)
**Interview's duration, min (mean ± SD)**	48 ± 5

### 4.3 Data collection

Data collection was performed through individual face‐to‐face interviews using a semi‐structured interview guide between one and three months after completion of the RCT study (Mourad et al., 2022). Participants had the opportunity to choose the time and place of the interview. Three interviews were conducted in participants’ homes and seven were conducted in a specific conversation room at the university, where the interviewer tried to create a calm, trusting and non‐judgmental atmosphere. Before the interviews were conducted, participants received information about the interview and the possibility to terminate their participation at any point during the process without stating a reason.

After completing the interview, both interviewers and participants sat down and spoke casually to gain a sense of the participant's state of mind before the meeting ended. The interview questions focused on the aim of the study, e.g: “Has your partner's non‐cardiac chest pain affected you? If so, in what way?” or “Has your partner's non‐cardiac chest pain meant any change in everyday life for you; if so, in what way?” Follow‐up questions were posed, e.g., “Interesting, can you tell me more about it? Please, can you expand on that a little bit more?” One pilot interview was conducted and did not result in any changes to the interview guide and was therefore included in the analysis. The study was conducted in southern Sweden between June 2018 and February 2020. The interviews lasted between 29 and 75 min and were conducted by AKK (3 interviews) and GM (7 interviews). Both interviewers are associated professors and registered nurses with long clinical experience in cardiac care and extensive experience in conducting qualitative studies. The interviewers had no previous care relation to the participants in this study. The two interviewers worked in closeness before and after the interviews and frequently discussed how to achieve a similar approach to facilitate the respondents´ explorative answers during the interviews and discussed if saturation was achieved. The interviews were audio‐recorded with participants' permission and transcribed verbatim by MEL. All interview data were handled confidentially, which means that no individual's identity can be recognized through the study.

### 4.4 Data analysis

The analysis was performed according to Patton's (Patton, 2015) guide for content analysis of qualitative data. The overview of steps in the data analysis process is presented in Table 2. The focus was to search for regularities in the data and for patterns to sort into categories. First, recordings were listened to in their entirety to ensure the accuracy of the transcripts. After that, the transcripts were repeatedly read to make their content known. Three interviews were selected, read and coded individually by all authors who afterwards mutually discussed findings and developed a coding scheme. Based on this coding scheme, MEL systematically coded the remaining transcripts. After encoding was completed, the codes were sorted into inductive emerging patterns that constructed sub‐categories and categories, which were mutually discussed, rearranged and finally approved by all authors. The software NVivo was used by MEL to gain an overview of the data and to manage data analysis. Results were strengthened and exemplified with quotations, which are drawn from the raw data and translated into English (Patton, 2015).

**TABLE 2:** Overview of steps in the analysis process.

1. Accuracy check	The accuracy of the transcription was checked about the recordings
2. Repeated reading	Each script was read several times to make the content known
3. Coding	Three interviews were selected, individually read and coded by all authors
4. Creation of a coding scheme	Mutual discussion of the findings and development of a coding scheme was performed
5. Systematic coding	The remaining transcripts were coded according to the coding scheme
6. Sorting of codes and creation of categories	All codes were sorted into inductive emerging patterns and created into sub‐categories and categories
7. Final approvement of category overview	The category overview was approved based on discussions to reach an agreement about the final creation of the findings

### 4.5 Ethical considerations

The study was approved by the Regional Ethical Review Board in Linköping, Sweden (code 2017/343‐31) and was conducted according to the Helsinki Declaration (WMA, 2013). The patients with whom the spouses lived were informed about the study's aim and approved their spouses' participation in this study. When the spouses were invited to the study, they were given oral and written information about the study's aim, that participation was voluntary and that they had a possibility to cancel or withdraw their participation without further consequences for their or their partners' care. They were also informed that all data would be handled confidentially. All spouses signed a written informed consent before the interviews were performed. The study information was repeated in connection to the interview.

### 4.6 Rigour

The consolidated criteria for reporting qualitative research (COREQ) was used to assure the trustworthiness of the study (Tong et al., 2007). The COREQ checklist ensures that all the necessary information about the study is reported or clarified.

Qualitative research seeks to increase and broaden the in‐depth understanding of a phenomenon (Patton, 2015). To ensure trustworthiness in this study, transferability, confirmability, credibility and dependability were used (Lincoln & Guba, 1985). The study data was collected until the start of the COVID‐19 pandemic, when face‐to‐face interviews were no longer possible, which could impact trustworthiness. We are aware of the small number of interviews and understand that this may be regarded to jeopardize the achievement of saturation during the data collection, which was finalized due to the COVID‐19 pandemic. However, the interviews were rich and very informative. As an alternative concept to the saturation concept, information power, proposed by Malterud et al., 2016), has been applied in our study. Information power suggests that the more relevant information the sample holds for the study's purpose, the fewer participants are required. This concept relies on several factors: the study's aim, the specificity of the sample, the quality of the dialogue and the analysis strategy (Malterud et al., 2016). Having the specific aim of exploring the phenomenon—living with a partner suffering from NCCP—contributes to establishing the credibility of the results. Still, the phenomenon investigated is complex and may not be fully explored in this study due to the number of interviews. However, the high quality of interviews (mean 48 min) contributes to rich data. In addition, following Patton's analytic strategy (Patton, 2015), including triangulation between all authors, mutually developing coding schemes, and identifying the authors' preunderstanding via reflection before and during analysis to minimize pre‐understanding's affecting the analysis and to ensure that all findings and interpretations were based on study data, the study's credibility and dependability were strengthened (Lincoln & Guba, 1985). Furthermore, the findings may be transferable to other samples and settings thanks to the clear and transparent description of the context, participants' characteristics (Table 1) and data analysis processes (Table 2). These detailed methodological descriptions may also strengthen the dependability of study findings. Taken together, all these aspects indicate that our findings have an information power that can be considered as trustworthy.

## 5 FINDINGS

The findings portray spouses' experiences of living with a partner suffering from NCCP. Three categories and seven subcategories were identified. The categories were: ‘a feeling of being neglected’, ‘a tension between hope and despair’ and ‘a threat to ordinary life’ (Table 3).

**TABLE 3:** Overview of findings.

Category	Subcategories
A feeling of being neglected	Being disregarded by healthcare professionals
	Being kept out of the loop by one's partner
A tension between hope and despair	Having faith and compassion
	Being frustrated and unprepared
A threat to ordinary life	Everyday tasks and economic challenges
	Diminished leisure activities
	Challenges in couple relationships

### 5.1 A feeling of being neglected

This category contains two subcategories portraying the spouse's feelings of ‘being disregarded by healthcare professionals’ and ‘being kept out of the loop by one's partner’. The subcategories describe the feeling of being ignored and excluded by professionals and feelings of being kept out of the loop by one's partner.

#### 5.1.1 Being disregarded by healthcare professionals

Experiencing being excluded by healthcare professionals was commonly expressed by the spouses. They said that healthcare professionals very often did not invite them to participate in the dialogue regarding the care of their partners. They described a feeling of not being acknowledged by the healthcare professionals, even when they sat beside their spouse. Spouses did not always receive answers to their questions or received short answers without a contextual description. Moreover, they experienced that they did not receive important information when they were unable to be present during visits to the hospital. When the partner later tried to recall what the doctor said about the state of health, they usually only shared a part of the information, which made it difficult for the spouses to grasp the entire situation. Spouses shared concerns that although they are expected to play an important role in their partner's healthcare and treatment, they are seldom involved in the plan or invited to participate when the information is provided.

I absolutely believe that as a family member, one should have the right to meet someone […] who could talk to me and say, 'Okay, you are in this situation now and practically you could do this, this and that, which could help him and in the long run, you as well.' (4)

#### 5.1.2 Being kept out of the loop by one's partner

Spouses experienced that the partners did not want to talk about their chest pain. Their partners were perceived as not wanting to worry their spouses, and therefore, often kept quiet about ongoing chest pain. Sometimes, their partners talked about the pain episode afterwards, but mostly they kept it to themselves. However, spouses experienced what they could feel when their partner was in pain. Body posture, facial expressions, impaired breathing, atypical movements, or sudden rest could reveal ongoing chest pain. Usually, the spouses choose not to show when they observed their partner is in pain and just waited it out.

I almost feel guilty that she hasn't said anything like that, so maybe I should have taken better care of her and asked a little more than I usually do. (3)

The participants did not talk about the iCBT program in which they were participating. Spouses were aware that their partner was working with the iCBT program, but they rarely knew or had any details about their progress. They saw their partner sitting down in front of a computer or tablet when working with the program or going out to exercise. However, the spouses said that the partner wished to manage the program in private. Some spouses experienced that their partners became annoyed when they received questions about the program or comments about their work efforts. Commonly they wanted to know more but did not want to insult the partner's integrity.

I was surprised when he said he had been involved and I said, 'That's great,' and then someday I stood there reading something while he was on the computer and said, 'But you haven't answered these things' and he replied, 'No, I haven't had the energy or felt like doing that' Then I noticed it was about some gymnastics, 'Did you do it?' 'No' 'Why not?' And he didn't answer and then I asked again and he just got annoyed so no, I don't care, so I stopped asking and then I left. (7)

### 5.2 A tension between hope and despair

This category contains two subcategories: ‘having faith and compassion’ and ‘being frustrated and unprepared’. The subcategories described mixed feelings of faith, compassion and support; the feeling of being unprepared to deal with the chest pain; and frustration with the situation, as described by spouses.

#### 5.2.1 Having faith and compassion

The spouses described that leaning on hope and faith to get through challenges had become a way of dealing with worries related to partners' chest pain. Spouses spoke about the benefits of gaining strength from religion. Praying made them feel as if they were making an effort, which counteracted feelings of powerlessness. Placing their worries on their faith gave them relief and helped them manage their fears.

I have a belief in higher powers, so it was natural for me to seek help from above […] It provides comfort and it gives someone to share the burden with, so it's calming in its own way. Somehow, it gives you the feeling that you're doing something, even though you can't directly explain its impact. But it definitely has a calming effect. (3)

Spouses believed that all healthcare contacts or treatments happened for a reason and were necessary. They believed that the sacrifices they made, such as accompanying their partner during late‐night visits to the emergency department or cancelling their plans, were necessary, even if it felt demanding. Spouses felt compassionate and empathetic and did not regret the relationship with their partners, despite challenging times. They believed and hoped that the challenges would strengthen their relationship, and a vision of a life without their partner made them appreciate it even more.

Another way of being compassionate and caring for their partners was by encouraging them to rest and by freeing them from daily tasks. Spouses supported their partners' decisions on how to manage their chest pain. Sometimes decisions regarded seeking advice from healthcare workers. In other cases, it was enough for the spouse to be calm, rational and present or to take a step back and give the partner space to manage their pain by themselves.

I'm supportive, actually and I can understand certain situations, but I don't try to influence or stress him out, or force him. (9)

Spouses did not want to interfere with their partner's participation in the iCBT program. They were positively surprised when their partner talked about the program because it confirmed that they were aware of the impact the chest pain had on them. Although they did not ask as much about the program, they encouraged and supported when their partner was working on it. Spouses could not always determine whether it was the content of the program that promoted changes in their partner's behaviour, however participation in this iCBT program was seen as positive and hopeful.

Yes, but she also seeks what could be and I believe she has learned a lot from being in the program about herself and about chest pain. She searches for a lot of information on her own when it comes to this and that regarding pain. So, she has learned a lot, everything is positive. Anything that improves is positive. (6)

#### 5.2.2 Being frustrated and unprepared

Spouses shared the impression that their partners were not being taken seriously, were referred to different healthcare providers and received unplanned and poorly coordinated care regarding their chest pain. They received no explanation for the cause of the chest pain and opposing recommendations regarding its management. Some spouses described anger towards healthcare professionals who were unable to treat their partner's symptoms. Moreover, they also experienced disappointment when healthcare was delayed and when their partner's chest pain was ignored. They described feelings of powerlessness and felt burdened by their partner's disappointment. This frustration also arose when they experienced a lack of strategies to manage their partner's chest pain, anxiety and feelings of vulnerability. Spouses emphasized the need to receive counselling and training to be able to support their partner in managing their chest pain.

Some kind of guidance might be helpful for family members, like ‘if you're a family member of a person in that situation, think about this and that.’ So, some type of knowledge that can be helpful, but exactly what it entails, whether it's about reading something online and getting tips from a web link, or if it involves regularly seeing a psychologist or seeking counseling and so on, it feels highly individual on my part, because I don't know what would be right or wrong. (3)

Spouses sometimes felt frustrated that their partner did not always share the same view as them and felt as if a burden were laid on their shoulders. The anger also stemmed from not being able to plan their own lives, as the partner's chest pain could unexpectedly appear at any time. Spouses felt frustrated that the chest pain had such a substantial impact on their family's lives. All in all, the frustration caused by this situation made the spouses feel hopeless and drained of energy.

I become very dejected, just this feeling of 'what am I doing and how long will it be like this and how should I think?' The emotions become very negative and it's challenging to maintain a positive spirit, I think […] Besides the anger and frustration towards the healthcare system, you become tired, you don't have the energy anymore. I don't know if it's because you worry and need to question so much, but emotionally, it has affected me in a way that I have felt low and depressed and sometimes I get angry at *****. He can't help having pain, but eventually, it becomes like a burden in some way. (4)

Spouses felt expectations were placed on them, even if they were not always stated explicitly; expectation which could come from their partner, relatives, healthcare professionals, or from themselves, which made them feel frustrated. Some of the expectations regarded practical home tasks such as cleaning and shopping and coordination of contacts with healthcare professionals. One of the most demanding and frustrating expectations spouses felt was conveying an understanding and empathic attitude towards their partner.

The expectations from [him] that one should understand it, are difficult. I mean, to understand how he feels, both in terms of his pain and how he feels in the whole situation, it's quite challenging because, well, it's really difficult, because you primarily prioritize your own perspective first of all (4)

Spouses felt confused and angry when witnessing their partners suffering from chest pain. Not being able to help or have anyone to contact made them feel abandoned and left them feeling like they were in a position of liminality.

Many times, I have sat and listened to ***** and felt so frustrated, thinking, 'What can I do as a relative?' Sometimes, I feel like I just wanted to, even though I know it's not possible, pick up the phone and say, 'Come on, let's have a meeting, let's talk because this isn't right. Why not just do it properly, check what you know and can check, so we can move forward because our whole family is stuck in neutral'. (4)

### 5.3 A threat to ordinary life

This category contains three subcategories: “everyday tasks and economical challenges”, “diminished leisure activities” and “challenges in couple relationships”. These subcategories describe participants' experiences of changes caused by chest pain and their negative impact on different aspects of their lives, such as everyday life and economy, leisure activities and couple relationships.

#### 5.3.1 Everyday tasks and economic challenges

Spouses' everyday lives were notably changed by the fact that the partners were limited in performing everyday tasks such as shopping, carrying bags, cleaning and making efforts at home. In some cases, this was also supported by healthcare professionals, who urged them to refrain from strenuous activities which spouses felt placed even higher demands on them. Spouses also experienced increased responsibility for the care of the children, as they felt the partner was not able to manage, in case of chest pain.

I'm afraid of not being there and being present if, for some reason, he doesn't feel like he can handle it on his own. And for my son, too, if ***** ends up in a state that he has no control over and my son has to witness it, that's the fear. And of course, I'm afraid that something might happen. (4)

The financial situation was another concern affected by the partners’ sick leave or having to quit work earlier, due to chest pain. Spouses' jobs were in some cases also affected by their partners' chest pain, or when they needed to support or relieve their partner from everyday tasks. This led to poorer finances that could impact their living conditions, in terms of possibilities to restore the house, go on vacations, or to get insurance or a bank loan.

It affects me when I have to travel for work. I may even hesitate, even if it's mandatory. My colleagues at work know the situation and I can take advantage of this and have a conversation with my boss. (4)

#### 5.3.2 Diminished leisure activities

Reduction of physical activity was pointed out by spouses as the biggest change in everyday life. Reduced movement and activity were experienced as related to episodes of chest pain, the fear of pain, or anxiety about the causes of pain. Physical activities that spouses had previously perceived as being enjoyed by partners were shortened and made less intense. Even their long joint walks were replaced by shorter walks or removed completely, which also affected spouses' everyday lives, as they also became more inactive and isolated at home.

I do the heaviest tasks in daily care here and then I try to encourage her to maybe go out for a walk. Somehow, I hope that movement will make things better. At least that's what I believe, so I try to accompany her (6)

They expressed the importance of having their activities and the opportunity to go out and leave difficulties related to chest pain behind. The spouses described the struggle to distance themselves from the chest pain and the consequences that came with it.

#### 5.3.3 Challenges in couple relationships

Spouses experienced a changed relationship. They were on guard and prepared to handle the worst‐case scenario, causing worries and anxiety over the idea that the partner suddenly could die and that life as they knew it could change dramatically. This was described as lying awake at night, anxious and listening to see whether their partner was still breathing.

My life has been filled with so much worry, so much anxiety about how ***** is doing. There are times when I'm barely able to leave the bedroom and I sneak in and listen. If I don't hear anything, I gently place my hand to feel if he's still breathing. (1)

The fear of causing chest pain also led to a changed sex life, and the relationship could transform from a desire for intimacy into camaraderie. The shift from being a partner to a caregiver changed the balance in the relationship. This was perceived as a threat because they wanted to be in a coupled relationship and not a caregiver to their partner. The spouses described sadness over not being able to socialize with others and do things together as a couple. They were aware that the pain was not their partner's fault, however, they felt anger, since the partner let the chest pain control their lives. They also felt sad for being stuck in an undesirable situation. They needed a break from the chest pain and sometimes questioned staying in the relationship.

There are days when you feel like it will never end and then these feelings arise, like, should you save yourself and go, or should you stay. (4)

## 6 DISCUSSION

In this study, we explored spouses´ experiences of living with a partner suffering from NCCP. Spouses’ experiences were categorized as “a feeling of being neglected”, “a tension between hope and despair” and “a threat to ordinary life”. There are several studies performed on spouses or families of patients with established medical diagnoses, such as heart failure (Imes et al., 2011; Kim et al., 2020), chronic pain (De Sola et al., 2023), chronic musculoskeletal pain (Smith et al., 2021), multiple sclerosis (Neate et al., 2019), multiple chronic conditions (Ploeg et al., 2020) and cancer (Lambert et al., 2012). However, this study differs in that it explores experiences of living with a partner with an unclear condition and recurring symptoms that are difficult to manage as they often do not receive a diagnosis or explanation for their symptoms, and therefore, fall between the cracks.

To begin with, spouses felt they were neglected and disregarded by healthcare by not being involved in the partners care, not getting answers on their questions and not being informed about their partners’ NCCP, which in some cases led to frustration. Even though they did not feel involved in planning their partner's support, they said that they were expected to play a crucial role in their partner's care. Similar findings have been reported by (Imes et al., 2011) who described partners' experiences of severe heart failure, as uninformed and left in the dark. Although there are similarities between the spouses of patients with NCCP and those with other medical diagnoses, one may consider that spouses of patients with NCCP are more vulnerable and lonelier since they have nowhere to turn to and no one to ask about their needs about their partner's chest pain, as the pain lacks an established diagnosis and therefore, they have no obvious care provider to turn to. This, on the contrary, is easier when the patient has a diagnosis and an established healthcare contact. A meta‐ethnographic review showed that spouses of heart failure patients had good relationships and were acknowledged as caregivers by healthcare professionals, and this gave them a feeling of engagement (Kim et al., 2020), but despite this, there is a need for increased acknowledgement and support (Kim et al., 2020; Smith et al., 2021). Moreover, spouses of patients with NCCP also said that they were kept out of their partners’ chest pain and how this was managed, including involvement in the iCBT program. It was a common belief that partners didn't want to share their worries or show that they were in pain, in order not to worry their spouses. Feeling disregarded and kept out of the loop when it came to their partners´ chest pain and care can impact spouses' abilities to support their partners. In spouses to multiple sclerosis patients, togetherness was described as a positive aspect which could mean working as a team and making important decisions together. This was perceived to create a bond and strengthen the couple's relationship (Neate et al., 2019). This suggests that in healthcare encounters it could be important to invite the spouses to let them be seen and heard, provide them with desired information and try to establish a collaboration between the couples in order to increase the sense of togetherness.

The spouses in our sample described their situation as bouncing back and forth between feelings of faith, compassion and support; being unprepared to deal with the chest pain and frustration with the situation, in general. Similar patterns have been described in other studies (Kim et al., 2020; Ploeg et al., 2020); where Ploeg et al. (Ploeg et al., 2020) reported that spouses of patients with multiple chronic conditions expected to be involved in their partner's care because they perceived this to be part of their marriage (Ploeg et al., 2020), whereas spouses in the study by (Kim et al., 2020) reported that seeing their loved ones deteriorate gave them a sense of fear and hopelessness. To cope with this, some spouses believed and hoped that the challenges would strengthen their relationship, while others leaned on religion to gain strength and to handle their fears and worries. Another way to go approach chest pain and the related worries was to remain positive and look for the best in situations, which also was described by (West et al., 2012). However, spouses stated that it was demanding to deal with the partner's chest pain when the feeling of hope was taken away.

Taken together, this suggests the importance of supporting the spouses in keeping faith and gaining the strength to be compassionate and supportive. Furthermore, spouses need help to become more prepared to deal with the complex situation of being in a relationship impacted by NCCP. This can be achieved by offering them counselling or training in pain management and dealing with worries caused by chest pain. In addition, spouses need advice on how to deal with economic challenges when their partner is on sick leave or retires early due to NCCP, something which the spouses in our study struggled with and has also been reported by (Smith et al., 2021). Several studies have pointed out the importance of knowledge and information regarding the care recipient's disease to regain control and increase their sense of satisfaction (Kim et al., 2020; Smith et al., 2021). Another aspect is helping spouses balance caregiving and everyday life (Kim et al., 2020). In our study, spouses reported taking on greater responsibility for household tasks and had less leisure time, which impacted their everyday lives negatively as they became inactivated and isolated at home. This is a common theme in other studies, in which spouses also reported social isolation and feeling constrained, even if they left home, as they were plagued by feelings of worry and guilt (De Sola et al., 2023; Ploeg et al., 2020). Although some spouses reported that the challenges of dealing with NCCP could strengthen their relationship, others experienced strained and changed relationships, e.g., a change in relationship roles and a loss of intimacy. In the worst cases, some questioned staying in the relationship.

To conclude, this study, among others, shows that spouses of patients with somatic problems, such as NCCP, have complex physically, emotionally and socially strained living situations and unmet needs (De Sola et al., 2023; Imes et al., 2011; Kim et al., 2020; Lambert et al., 2012; Neate et al., 2019; Ploeg et al., 2020; Smith et al., 2021). It is important that healthcare professionals, including nurses, do not forget spouses in their encounters with NCCP patients and acknowledge and support them in their struggle to help their partners to deal with chest pain and thereby not become healthcare consumers themselves. We therefore believe that nurse‐led interventions focusing on information, education and psychosocial support could help spouses feel informed and prepared to handle their situation in relation to NCCP and give them tools to handle negative thoughts and emotions and regain control over their own lives.

### 6.1 Strength and limitations

The number of participants may be seen as a limitation. Yet, since data collection was conducted through face‐to‐face interviews, it was stopped due to the COVID‐19 pandemic. On the other hand, the study can be seen to have sufficient information power, as described by (Malterud et al., 2016), since all participants had personal experience of living with a partner struggling with NCCP and thus contributed valuable knowledge to the study. In addition, the interview guide focused on the study aim and it can be regarded as a strength that the majority of the data was relevant to the analysis in the present study. Furthermore, the interviews were conducted by experienced interviewers and had an average duration of 48 minutes, indicating rich data, which were analysed with an established analytic strategy according to Patton (Patton, 2015). To increase trustworthiness, triangulation was performed during the data analysis, which strengthened the accuracy of interpretations and study findings.

### 6.2 Implications for policy and practice

This study is relevant to clinical practice as it provides insights into the experiences of spouses living with partners suffering from NCCP. One important finding is that spouses of patients with NCCP often feel neglected by both their partners and healthcare professionals, despite their important roles in their partners' lives. Nurses should acknowledge and promote the involvement of partners in managing their loved ones' conditions. Another finding is that spouses often feel unprepared to address their partners' chest pain. An intervention focused on information, education and psychosocial and practical support could equip spouses with the tools they need to better handle the daily challenges associated with NCCP. Additionally, spouses require support to effectively manage negative thoughts and emotions and regain control over their own lives. In essence, this study's findings not only illuminate the nuanced experiences of spouses dealing with NCCP but also underscore the need for tailored interventions and increased recognition of the central role spouses play in their partners overall well‐being.

### 6.3 Recommendations for further research

Further research should aim to follow a cohort of couples over an extended period to observe changes in their emotional experiences and practical challenges as they adapt to living with NCCP. This endeavour aims to provide insights into the long‐term effects and coping mechanisms associated with NCCP. Comparing the emotional and practical effects of chest pain between spouses of patients with NCCP and spouses of patients with cardiac conditions may also reveal unique challenges specific to NCCP and offer a broader perspective on the overall impact of chest pain. Exploring the influence of cultural backgrounds and gender roles can shed light on how distinct cultural norms and gender dynamics shape the emotional and practical impact on spouses. This aspect of the study may reveal nuances in how individuals from different backgrounds respond to and deal with the challenges posed by NCCP. Finally, we propose the development and testing of interventions designed to inform, educate and support spouses in managing both the practical and emotional aspects of their daily lives. In addition, considering an intervention aimed at improving communication and mutual support within couples where one partner has NCCP holds promise. This intervention may involve components such as psychoeducation, counselling, or participation in support groups, all of which aim to empower couples to jointly deal with the challenges related to NCCP.

## 7 CONCLUSIONS

NCCP in partners had an emotional and practical impact on the lives of spouses. The spouses described feelings of being neglected and uninvited by nurses and their partners. The struggles in everyday life and couple relationships created a tension between hope and despair where faith and hope met powerlessness and frustration over the situation posed by NCCP. The findings suggest that nurses should also pay attention to the needs of the spouses when encountering patients with NCCP. This may facilitate spouses to at least feel seen and heard during the caring encounter. However, interventions need to be developed to strengthen the well‐being of the spouses and to support them in managing the struggles of everyday life with NCCP.

## AUTHOR CONTRIBUTIONS

MEL, PJ and GM contributed to the conception and design of the study. AKK and GM performed the interviews. MEL transcribed the interviews. MEL, AKK, PJ and GM processed the data and performed the qualitative analyses and drafting of the manuscript.

## FUNDING INFORMATION

The study was funded by the Kamprad Family Foundation (number ISV‐2018‐00033).

## CONFLICT OF INTEREST STATEMENT

The authors declare that they have no conflict of interest.

## ETHICS STATEMENT

The study was conducted according to the Helsinki Declaration and was approved by the Regional Ethical Review Board in Linköping, Sweden (code 2017/343‐31). The patients with whom the spouses lived approved of their spouses' participation in this study. All spouses were given oral and written information about the aim of the study and signed a written informed consent to participate in the study before the interviews were performed. They were also informed about the possibility of cancelling or withdrawing their participation in the study.

## CONSENT FOR PUBLICATION

Not applicable.

## TRIAL REGISTRATION

The study was registered on 01/11/2017 at ClinicalTrials.gov NCT03336112; https://www.clinicaltrials.gov/ct2/show/NCT03336112

## Data Availability

The datasets used and/or analysed during the current study are available from the corresponding author on reasonable request.
